# Marine RNA Virus Quasispecies Are Distributed throughout the Oceans

**DOI:** 10.1128/mSphereDirect.00157-19

**Published:** 2019-04-03

**Authors:** Marli Vlok, Andrew S. Lang, Curtis A. Suttle

**Affiliations:** a Department of Botany, University of British Columbia, Vancouver, British Columbia, Canada; b Institute for the Oceans and Fisheries, University of British Columbia, Vancouver, British Columbia, Canada; c Department of Biology, Memorial University of Newfoundland, St. John’s, Newfoundland and Labrador, Canada; d Department of Earth, Ocean and Atmospheric Sciences, University of British Columbia, Vancouver, British Columbia, Canada; e Department of Microbiology and Immunology, University of British Columbia, Vancouver, British Columbia, Canada; DOE Joint Genome Institute; Oregon State University; Japan Fisheries Research and Education Agency

**Keywords:** *Picornavirales*, biogeography, marine RNA virus, purifying selection, quasispecies

## Abstract

RNA viruses, particularly genetically diverse members of the *Picornavirales*, are widespread and abundant in the ocean. Gene surveys suggest that there are spatial and temporal patterns in the composition of RNA virus assemblages, but data on their diversity and genetic variability in different oceanographic settings are limited. Here, we show that specific RNA virus genomes have widespread geographic distributions and that the dominant genotypes are under purifying selection. Genomes from three previously unknown picorna-like viruses (BC-1, -2, and -3) assembled from a coastal site in British Columbia, Canada, as well as marine RNA viruses JP-A, JP-B, and Heterosigma akashiwo RNA virus exhibited different biogeographical patterns. Thus, biotic factors such as host specificity and viral life cycle, and not just abiotic processes such as dispersal, affect marine RNA virus distribution. Sequence differences relative to reference genomes imply that virus quasispecies are under purifying selection, with synonymous single-nucleotide variations dominating in genomes from geographically distinct regions resulting in conservation of amino acid sequences. Conversely, sequences from coastal South Africa that mapped to marine RNA virus JP-A exhibited more nonsynonymous mutations, probably representing amino acid changes that accumulated over a longer separation. This biogeographical analysis of marine RNA viruses demonstrates that purifying selection is occurring across oceanographic provinces. These data add to the spectrum of known marine RNA virus genomes, show the importance of dispersal and purifying selection for these viruses, and indicate that closely related RNA viruses are pathogens of eukaryotic microbes across oceans.

**IMPORTANCE** Very little is known about aquatic RNA virus populations and genome evolution. This is the first study that analyzes marine environmental RNA viral assemblages in an evolutionary and broad geographical context. This study contributes the largest marine RNA virus metagenomic data set to date, substantially increasing the sequencing space for RNA viruses and also providing a baseline for comparisons of marine RNA virus diversity. The new viruses discovered in this study are representative of the most abundant family of marine RNA viruses, the *Marnaviridae*, and expand our view of the diversity of this important group. Overall, our data and analyses provide a foundation for interpreting marine RNA virus diversity and evolution.

## Abstract (precis)

Very little is known about aquatic RNA virus populations and genome evolution. This is the first study that analyzes marine environmental RNA viral assemblages in an evolutionary and broad geographical context. This study contributes the largest marine RNA virus metagenomic data set to date, substantially increasing the sequencing space for RNA viruses and also providing a baseline for comparisons of marine RNA virus diversity. The new viruses discovered in this study are representative of the most abundant family of marine RNA viruses, the *Marnaviridae*, and expand our view of the diversity of this important group. Overall, our data and analyses provide a foundation for interpreting marine RNA virus diversity and evolution.

## INTRODUCTION

Viruses shape the ecology and evolution of marine microbial communities. They are the most abundant biological entities in the ocean and are estimated to kill about 20% of its living biomass each day, thus affecting food web dynamics and biogeochemical cycling (1, 2). Most of these inferences are based on studies of double-stranded DNA (dsDNA) viruses, primarily phage. Yet, increasing evidence shows that RNA viruses are also important players in the ecology of marine ecosystems (3).

The first evidence that RNA viruses are pathogens of marine phytoplankton was the isolation of a picorna-like virus that infects the toxic, bloom-forming microalga Heterosigma akashiwo (4) and subsequent environmental sequencing that established the enormous diversity in seawater of viruses in the order *Picornavirales*. Targeted sequencing of picorna-like virus RNA-dependent RNA polymerase (RdRp) genes revealed that most sequences fell into a distinct phylogenetic cluster, which are likely viruses primarily infecting protists (5–9). An ensuing metagenomic survey of RNA viruses demonstrated that sequences associated with picorna-like viruses were the most abundant reads that could be assigned (10).

Not only are the abundance and diversity of viruses in the oceans high, but there are patterns in their distribution. Although identical or nearly identical viral genotypes have been documented in geographically and environmentally separate locations (6, 11–13), which is consistent with viral dispersal across vast distances (14), other data show that biogeographical patterns occur in some groups of marine DNA viruses (15). The global distributions of viruses are influenced by virus life cycle (i.e., lytic versus lysogenic/latent), replication rate, length of the latent period, burst size, and the presence of host-derived genes (16, 17) and atmospheric circulation (14). RNA viruses infecting protists are small, with lytic life cycles and large burst sizes and lacking auxiliary metabolic genes (18). For example, the diatom-infecting Rhizosolenia setigera RNA virus (RsRNAV) (19) and Chaetoceros tenuissimus RNA virus 01 (CtenRNAV01) (20) have different life cycles, which should affect their relative distributions. RsRNAV has a smaller burst size (10^3^ versus 10^4^) and a longer infection cycle (2 days versus <24 h), so it should have a lower distribution potential.

Being obligate parasites, the success of virus genotypes (i.e., population growth) is tightly coupled with the virus hosts. Host traits, such as abundance, distribution, size, and physiological state, affect the biogeography of their viruses (16). Strain specificity will also potentially affect distribution, as a broader host range yields more potential hosts. RsRNAV, CtenRNAV01, Heterocapsa circularisquama RNA virus (HcRNAV) and Heterosigma akashiwo RNA virus (HaRNAV) infect widely distributed taxa, although the viruses are also strain specific (4, 19–21), which reduces the population size of potential host cells and, therefore, the virus distribution potential. Nonetheless, infectious HaRNAV in coastal sediments (22) and the presence of viruses JP-A and JP-B at multiple locations in the coastal waters of British Columbia (23) imply that some viruses are temporally and spatially widespread.

Virus-host coupling and infection specificity drive a coevolutionary arms race (24). RNA viruses are well adapted for this by having high mutation rates due to their error-prone RdRp, which results in approximately 10^−4^ substitutions per nucleotide per replication cycle (25, 26). Thus, a single progenitor generates a distribution of genetically diverse genotypes, a quasispecies, that is clustered around the fittest sequence (27–29). Quasispecies are thought to provide virus populations with built-in flexibility to overcome host and environmental challenges (30–32). While quasispecies are well characterized in RNA viruses associated with higher plants and mammals (33–35), environmental quasispecies are largely unexplored. An analysis of quasispecies in assembled RNA virus genomes from an Antarctic lake suggested that the ecological setting was important in selecting the fittest genotype, as more single-nucleotide variants (SNVs) occurred in lake water than in microbial mats (36). Whether this variability was due to a greater diversity and turnover or because water from multiple locations mixes in the lake is unclear.

Selection ultimately determines which quasispecies genomes are most successful. Under neutral selection, a simple relationship exists between the rates at which mutations are generated and established within a population (37); deviations from neutral selection reveal evolutionary processes such as natural selection (38). For example, RNA viruses in plants that are transmitted by arthropod vectors exhibit lower rates of nonsynonymous (*dN*) changes than synonymous changes (*dS*), indicating elevated purifying (or negative) selection pressure compared to that of viruses transmitted by other routes, probably due to the required interactions of virus capsids with insect cellular receptors (30). In contrast, influenza A viruses break periods of evolutionary stasis with intervals of positive selection, where *dN* exceeds *dS*, rapidly replacing circulating lineages with new ones, allowing the virus to escape immune pressure within a host population (39). Marine RNA viruses infecting protists have different obstacles than viruses infecting multicellular organisms. While purifying selection has been proposed for the RdRp of marine picorna-like viruses (40, 41), there is little understanding of the evolutionary pressures facing these viruses or how they respond to them. The known marine single-stranded RNA (ssRNA) viruses are important from both an ecological perspective and a larger evolutionary context. These viruses are part of an order (the *Picornavirales*) of viruses with ancient evolutionary origins and phylogenetic primacy among modern RNA viruses (42, 43). They are also abundant in seawater, with their numbers potentially rivaling those of DNA viruses (3).

Here, we examine six marine RNA viruses in the context of biogeography, evolutionary patterns, and quasispecies potential. HaRNAV, a cultivated virus, infects the toxic bloom-forming raphidophyte Heterosigma akashiwo (4), has a 9.1-kb monocistronic genome (44), and is the type species of the family *Marnaviridae* (45). JP-A and JP-B were assembled from metagenomic data, are dicistronic, have 9.2-kb and 8.8-kb genomes, respectively, and based on phylogenetic analysis of RdRp domain sequences, belong within a well-supported clade of marine picorna-like viruses (23); it is believed that these viruses infect protists (9). The other three virus genomes were assembled in this study from metagenomic data. The biogeographic patterns of these six viruses were analyzed across 15 locations, including the Arctic, the Antarctic, the west coasts of North and South America, the Gulf of Mexico, Hawaii, the southern coast of South Africa, two freshwater lakes, and reclaimed water. Based on analysis of SNVs, our results show that these viruses exist as quasispecies and display biogeographical patterns in their distribution.

## RESULTS

Three major findings stem from the results presented here. First, we assembled three previously unknown positive-sense ssRNA viruses from the coastal waters of British Columbia. Second, recruitment analysis of assembled metagenomic data collected across many distant marine and freshwater locations to these genomes, and three others from the same area, demonstrates that close relatives of these viruses occur worldwide. Finally, a detailed analysis of the sequence variation found across locations is consistent with biogeographic patterns and production of quasispecies within these viruses. These results are presented in detail below.

### Assembly of novel marine picorna-like viruses.

**FIG 1:**
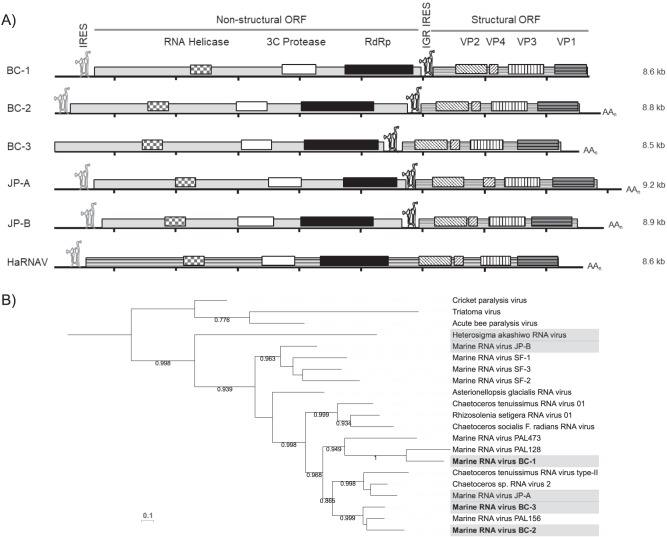
Three nearly complete viral genomes assembled from the Jericho Pier sample. (A) The genomes display the quintessential genome composition common to members of the order *Picornavirales*. The 5′ ORFs contain the nonstructural protein domains (RNA helicase [checkered rectangles], 3C protease [white rectangles], and RNA-dependent RNA polymerase [RdRp] [black rectangles]), and the 3′ ORFs contain the four structural protein domains (striped rectangles) and are preceded by predicted intergenic region (IGR) IRES elements. The four capsid domains are organized similarly to those of HaRNAV, with VP2 at the 5′ end, followed by VP4, VP3, and VP1. (B) A maximum likelihood phylogeny of the RdRp gene. The six genomes used in this analysis are highlighted in gray. The three dicistrovirus type species were used as an outgroup.

Three previously unknown and nearly complete picorna-like virus genomes (BC-1, BC-2, and BC-3) were assembled from metagenomic data collected at Jericho Pier in April 2014 (JP14) (Fig. 1). The viruses were dicistronic, with 8,638-nucleotide (nt) (BC-1), 8,843-nt (BC-2), and 8,496-nt (BC-3)-long genomes and GC contents of 41.2, 42.3, and 43.4%, and were assembled from 11,100, 31,880, and 156,011 reads with coverages of 128.65, 359.36, and 1,816.97 times, respectively. Seven well-conserved motifs were identified in each genome. The 3′ end of marine RNA virus BC-1 and the 5′ end of BC-3 were not complete, as judged by the lack of untranslated regions (UTRs) (Fig. 1). Poly(A) tails were identified at the 3′ ends of the BC-2 and BC-3 genomes after UTRs of 315 and 273 nt, respectively, indicating that these regions were completely sequenced. The 5′ UTRs of BC-1 and BC-2 were 634 and 247 nt, respectively and contained putative internal ribosome entry site (IRES) elements. The closest genetic relatives in the NCBI nonredundant database to the three novel viruses were all assembled genomes from metagenomic studies with no host information available. BLASTx analyses of the open reading frames (ORFs) indicated that both ORFs of BC-2 and -3 were most similar to marine RNA virus PAL 156 with E value scores of 0.0 and amino acid identities of 59% and 53% (nonstructural ORFs) and 59% and 60% (structural ORFs), respectively. The BC-1 virus exhibited the highest similarity to marine RNA virus PAL 128, with E values of 0.0 for both ORFs by BLASTx and amino acid identities of 51% and 45% for the nonstructural and structural ORFs, respectively. These viruses were used for all subsequent analyses, along with JP-A and JP-B, which also originated from Jericho Pier (6), and HaRNAV, which was isolated from the nearby Fraser River plume (4).

### Recruitment of assembled metagenomic reads to marine RNA virus genomes.

The geographical distributions of the six viruses were explored using 17 RNA virus metagenomic data sets (15 generated in the present study and 2 from previous studies). Library size potentially affects the detection of rarer viral genomes, but there was no trend between the number of reads recruited to genomes and library size (see Fig. S1 in the supplemental material), suggesting that this was not an issue for our data. The recruitment analysis was not competitive, meaning that a single read could theoretically be recruited up to six times, once to each genome. However, on average, each read that was recruited from a metagenomic data set to a genome was recruited only 1.0 to 1.5 times. Data from Palmer Station and Nunavut 3 were exceptions (Fig. S1) to the observed trend. Overall, our analysis indicates that the same reads did not map to all six genomes in each analysis.

10.1128/mSphereDirect.00157-19.3 FIG S1 Comparison of metagenomic library size and number of reads that mapped to the six genomes. Each library/location is represented by a different color, with the size of the circle denoting the mean of how many times reads were represented more than once/how often reads mapped to more than one genome on average. Download FIG S1, EPS file, 0.10 MB. Copyright © 2019 Vlok et al. 2019 Vlok et al. This content is distributed under the terms of the Creative Commons Attribution 4.0 International license.

**FIG 2:**
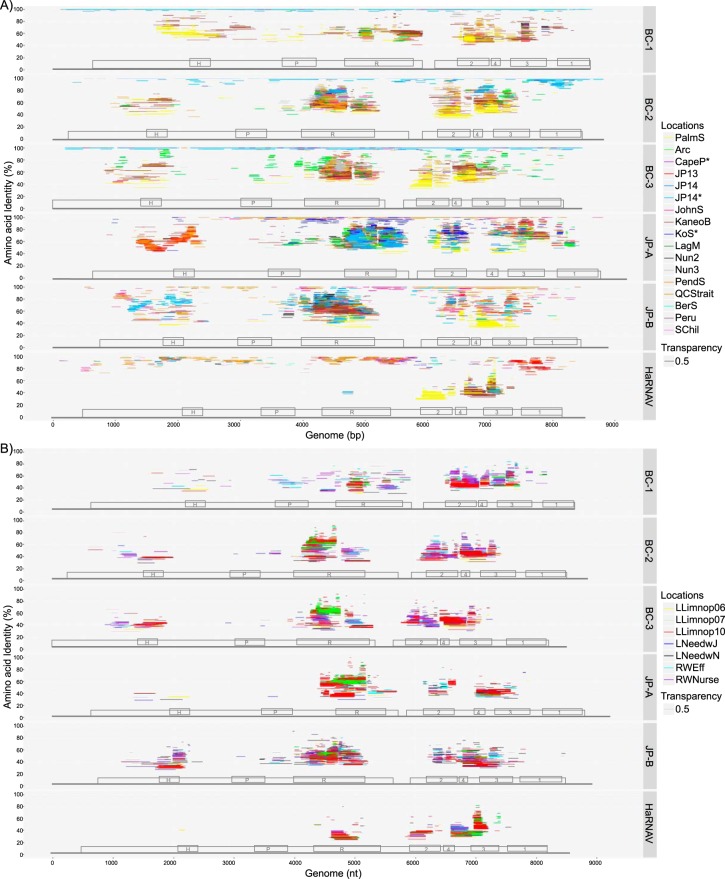
Sequences mapping to the six marine RNA viruses in the environmental viral metagenomic data sets. Fragments were recruited from marine RNA virus metagenomic data sets that were either generated during this study (Arctic [Arc], Cape Point [CapeP], Jericho Pier 13/14 [JP], Johnstone Strait [JohnS], Kenton-on-Sea [KoS], Laguna Madre [LagM], Nunavut 2/3 [Nun], Pendrell Sound [PendS], Queen Charlotte Strait [QCStrait], Bering Sea [BerS], Peru, and South Chile [SChil]) or from one of the publicly available metagenomic data sets, namely, Palmer Station (PalmS; Antarctica) or Kaneohe Bay (KaneoB; Hawaii) (A) or were from three freshwater data sets from Lake Limnopolar (LLimnop; Antarctica), Lake Needwood (LNeedw; MD, USA), and reclaimed water from Florida (RWEff and RWNurse; USA) (B). Metagenomic reads were recruited against each of the six virus genomes using tBLASTx with an E value of 10*^−^*^10^. The position of the colored bars on the *y* axis indicates the percent amino acid sequence identity to the virus, while the width of the bar covers the region of the genome where the similarity was observed. The bars are displayed at 0.5 transparency so that overlap among locations can be observed. Genome maps for each virus are superimposed on the plot with the domains labeled as follows: H, helicase; P, protease; R, RNA-dependent RNA polymerase; and 1, 2, 3, and 4, capsid VP1, VP2, VP3, and VP4, respectively.

**FIG 3:**
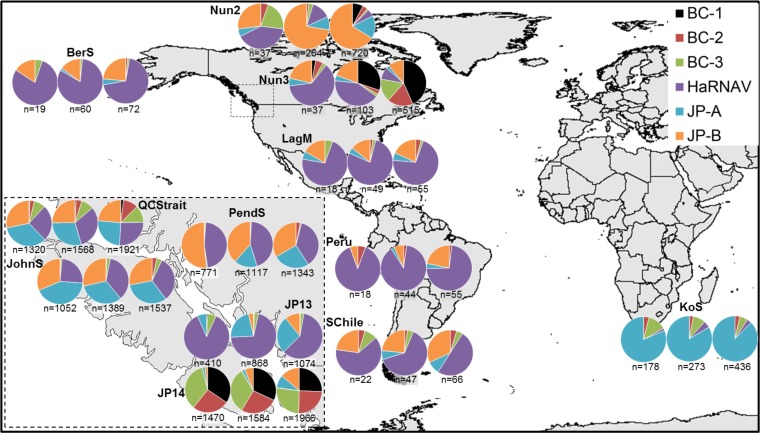
Distribution of six marine RNA virus genomes. Charts represent the number of reads recruited to each genome at 95, 85, and 75% protein identity with the number of total reads recruited below each chart. Genomes are represented by colors for marine RNA viruses BC-1 (black), BC-2 (red), BC-3 (green), JP-A (blue), JP-B (orange), and Heterosigma akashiwo RNA virus (purple). Metagenomic data sets were selected based on high percentage amino acid identity read recruitment to the six viruses and include the Bering Sea (BerS), Nunavut 2/3 (Nun), Laguna Madre (LagM), Peru, South Chile (SChil), Kenton-on-Sea (KoS), Queen Charlotte Strait (QCStrait), Pendrell Sound (PendS), Johnstone Strait (JohnS), and Jericho Pier 13/14 (JP).

To assess the distribution of close relatives of the six marine RNA viruses, reads were recruited to their genomes from 17 marine and 7 fresh and reclaimed water RNA virus metagenomic data sets collected from widespread locations (Fig. 2; Fig. S2 and S3). The amino acid similarity and distribution of metagenomic reads across the genomes varied markedly among locations and for each virus. Even for data sets collected from the location from which the virus was identified, detection of reads mapping to the genomes was sporadic; however, for the viruses BC-1, BC-2, and BC-3, identical reads (Fig. 2A) were assembled from the two JP14 data sets. In contrast, the JP14 samples yielded very few reads matching HaRNAV (Fig. 2A and 3; Fig. S3O and S3P), while for JP-A and JP-B they ranged between 40 and 90% identity and were predominantly within the RdRp domain. In contrast, many reads from the JP13 sample were similar to those from HaRNAV (80 to 98% amino acid identity), while few matched JP-A, JP-B, or BC-3 (Fig. S3Q).

10.1128/mSphereDirect.00157-19.4 FIG S2 Prevalence of reads with amino acid sequence identities greater than 85% mapped to the six marine RNA viruses. Fragments were recruited from marine RNA virus metagenomic data sets generated during this study (Arctic, Cape Point, Jericho Pier 13/14, Johnstone Strait, Kenton-on-Sea, Laguna Madre, Nunavut 2/3, Pendrell Sound, Queen Charlotte Strait, Bering Sea, Peru, and South Chile) (A) and from publicly available metagenomics data sets from Palmer Station (Antarctica) and Kaneohe Bay (Hawaii) (B). Metagenomic reads were recruited against each of the six virus genomes using tBLASTx with an E value of 10*^−^*^10^. The position of the colored bars on the *y* axis indicates the percentage amino acid sequence identity of the read to the virus, while the width of the bar covers the region of the genome where the similarity was observed. The bars are displayed at 0.5 transparency to make overlap more evident. Each genome map for each virus is superimposed on the plot with domains with designations from left to right as follows: helicase, protease, RNA-dependent RNA polymerase, VP2, VP4, VP3, and VP1. Download FIG S2, EPS file, 0.1 MB. Copyright © 2019 Vlok et al. 2019 Vlok et al. This content is distributed under the terms of the Creative Commons Attribution 4.0 International license.

10.1128/mSphereDirect.00157-19.5 FIG S3 Reads mapping to six marine RNA viruses represented by metagenome. Reads from Antarctica (A), the Arctic (B), Bering Sea (C), Cape Point (D), Chile (E), Queen Charlotte Strait (F), Peru (G), Pendrell Sound (H), Nunavut 3 (I), Nunavut 2 (J), Laguna Madre (K), Kenton-on-Sea (L), Kaneohe Bay (M), Johnstone Strait (N), Jericho Pier 14 (O, P), and Jericho Pier 13 (Q) mapped to marine RNA viruses BC-1, BC-2, and BC-3, JP-A, JP-B, and Heterosigma akashiwo RNA virus (HaRNAV). The superimposed domains are from left to right: helicase, protease, RNA-dependent RNA polymerase, VP2, VP4, VP3, and VP1. Download FIG S3, EPS file, 0.9 MB. Copyright © 2019 Vlok et al. 2019 Vlok et al. This content is distributed under the terms of the Creative Commons Attribution 4.0 International license.

Nonetheless, deduced amino acid sequence with high levels of identity to the six viruses were detected at numerous locations (Fig. 2A; Fig. S2). For example, JP-A was detected at 95 to 100% identity in samples from Queen Charlotte Strait (QCStrait) and Johnstone Strait (JohnS) (Fig. 2A and 3; Fig. S2, S3F, and S3N). The coverage spanned more than 3 kb of the genome (3,000 to 7,800 nt from QCStrait and 3,000 to 6,000 nt from JohnS). Similarly, at Johnstone Strait, 2 kb of the JP-B genome was covered by reads with 100% amino acid identity. These locations also had reads corresponding to BC-2, BC-3, and HaRNAV, but these were fewer than for the JP viruses and more sporadically distributed across the genomes. Reads with 80 to 100% amino acid identities to HaRNAV were detected in the Peru, Nunavut, Bering Sea, Laguna Madre, and South Chile samples (Fig. 3). Like HaRNAV, reads mapping to JP-A occurred beyond coastal British Columbia, and 60 to 100% amino acid identities were detected in the Kenton-on-Sea (KoS) metagenome from South Africa (Fig. S3L).

Many reads in the JP14 sample mapped with high identity to BC-1, -2, and -3; however, there was wide variation in identity levels across the genomes (Fig. 2A; Fig. S2). The pattern is exemplified by the VP1 (capsid protein) domain region of BC-2, where many sequences that mapped to this small region differed by as much as 15% amino acid identity (Fig. S2). A similar pattern was observed for the HaRNAV genome in the JP13 sample, where a large number of sequences also mapped to the VP1 domain, although the range in amino acid sequence identities was even greater.

Across all locations, a large proportion of the reads showed low levels of amino acid sequence identity to the six viruses, ranging from about 20% to 80% across both structural and nonstructural domain regions, although many reads mapped to RdRp domains. BC-1 and HaRNAV were exceptions, and fewer environmental reads mapped to their genomes overall (see Fig. 5). Generally, of the seven recognizable domains, the protease and VP1 regions had the fewest identified environmental reads.

Many reads from the assembled metagenomic data obtained from the five lakes and two reclaimed water sites recruited to the six marine RNA virus genomes, some with identities as high as 90%, although most were between 30 and 60% (Fig. 2B). As in the marine recruitment analysis (Fig. 2A), most of the reads recruited to regions associated with the helicase, RdRp, and VP-2, -3, and -4 domains.

### Domain-specific nucleotide variation.

**FIG 4:**
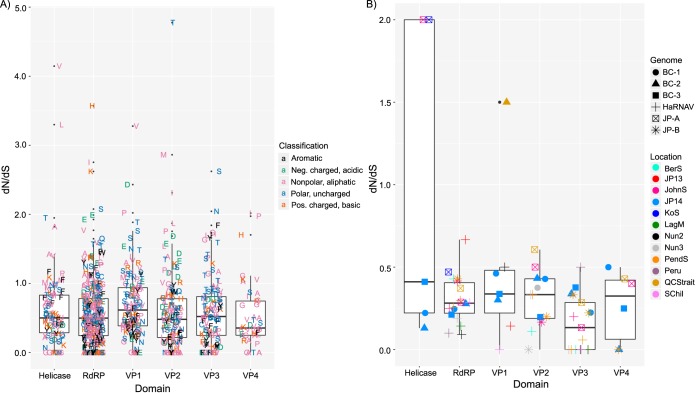
Selection analyses of marine RNA virus domains in environmental data sets. (A) Maximum likelihood analysis of selection pressure based on the codons of marine RNA viruses BC-1, BC-2, BC-3, JP-A, JP-B, and HaRNAV. The substitutions were estimated using the joint maximum likelihood reconstructions of ancestral states under a Muse-Gaut model (86) of codon substitution and the Felsenstein 1981 model (87) of nucleotide substitutions. Amino acids were classified as aromatic (phenylalanine [F], tryptophan [W], tyrosine [Y]), negatively charged (–), acidic (aspartic acid [D], glutamic acid [E]), nonpolar, aliphatic (alanine [A], glycine [G], isoleucine [I], leucine [L], methionine [M], proline [P], valine [V]), polar, uncharged (asparagine [N], cysteine [C], glutamine [Q], serine [S], threonine [T]), and positively charged (+), basic (arginine [R], histidine [H], lysine [K]). (B) Selection pressure acting on the various domains of the six marine RNA viruses at various locations. Single-nucleotide variants (SNVs) were called using the quality-based variant detection tool (CLC Genomics Workbench) from alignments in which environmental sequences with ≥90% sequence identity were mapped to the genomes. The helicase domain was not analyzed due to insufficient data. Two additional virus domain-sample pairings were omitted due to insufficient data (VP1 of both BC-2 and -3 from the Nun2 and QCStrait samples, respectively).

In order to infer selection pressures on the viral genomes, we examined nucleotide substitutions in six of the conserved domains for each of the viruses. Based on their consensus sequences, all of these domains were subject to purifying or negative selection (Fig. 4A). Values of *dN*/*dS* of <1.0 for all data below the 75th percentile indicates that purifying selection was acting on these sequences. No difference was observed among the calculated means for the six domains (analysis of variance [ANOVA], *P* = 0.2507), but based on the medians, all groups did not have the same distributions of *dN*/*dS* ratios (Kruskal-Wallis rank sum test, *P* = 0.0385). Multiple pairwise comparisons showed that the VP1 domain had a statistically significant larger median *dN*/*dS* ratio distribution than the RdRp and VP2 domains (Conover-Iman test; *P* = 0.0364 and 0.0442, respectively) (Table S2). Several codons were under neutral selection and a few more were under positive selection, indicating that the latter sites are more variable. All groups of amino acids (aromatic, acidic, basic, aliphatic, and uncharged) were subject to the same evolutionary pressure (Fig. S4).

10.1128/mSphereDirect.00157-19.6 FIG S4 The number of amino acid groups per *dN*/*dS* ratio based on the calculated conserved amino acid sequences of the six marine RNA virus genomes. Domains are, from left to right, helicase, protease, RNA-dependent RNA polymerase, VP2, VP4, VP3, and VP1. Download FIG S4, EPS file, 0.06 MB. Copyright © 2019 Vlok et al. 2019 Vlok et al. This content is distributed under the terms of the Creative Commons Attribution 4.0 International license.

Selection analyses of the metagenomic data that mapped to the six genomes reflected purifying selection acting on these viruses (Fig. 4B). There were no significant differences among the domains (ANOVA, *P* = 0.05343; Kruskal-Wallis rank sum, *P* = 0.09858). Calculated *dN*/*dS* ratios were very low apart from outliers for the VP1 domain of virus BC-2 in Queen Charlotte Strait and the helicase domain of JP-A in Johnstone Strait and Kenton-on-Sea.

**FIG 5:**
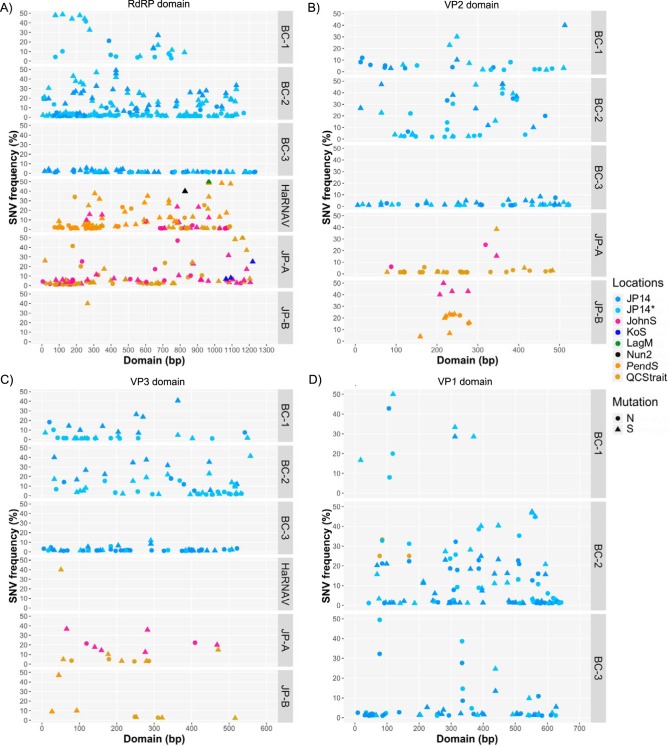
Detection of marine RNA virus quasispecies in environmental data sets. The distribution of single*-*nucleotide variants (SNVs) along the RdRp (A), VP2 (B), VP3 (C), and VP1 (D) domains of marine RNA viruses BC-1, BC-2, BC-3, HaRNAV, JP-A, and JP-B. Triangles (▲) indicate synonymous mutation variants, and circles (●) indicate nonsynonymous variants. The locations/samples where variants were detected are illustrated in different colors and include Jericho Pier (JP; light blue), Johnstone Strait (JohnS; pink), Kenton-on-Sea (KoS; royal blue), Laguna Madre (LagM; green), Nunavut 2 (Nun2; black), Pendrell Sound (PendS; gold), and Queen Charlotte Strait (QCStrait; brown).

Single-nucleotide-variant (SNV) analyses (Fig. 5) indicated that clouds of diverse variants for each of the six marine viruses existed in different samples. Because two sequence libraries were prepared from the Jericho Pier 14 sample, and most SNVs occurred in both libraries, this suggests that sequencing errors were not a significant source of the variation. Sequences mapping to viruses BC-1 and -2 were abundant in the Jericho Pier 14 sample, and many SNVs were distributed across the RdRp, VP2, and VP3 domains, at frequencies of 1 to 50% (Fig. 5A, B, and C). Most variants were synonymous, except in the VP2 domain, which had more nonsynonymous mutations. In contrast, SNVs in the same three domains of BC-3, as well as in its helicase domain, occurred at much lower frequencies (1 to 12%). Though fewer SNVs were observed across the domains of BC-1 and -2, these SNVs occurred more often within the quasispecies, resulting in a higher frequency of occurrence (Fig. S5A). The VP1 domain of BC-3 exhibited a similar trend of low-frequency mutations, except for three variants that occurred at considerably higher frequencies, two of which were nonsynonymous. Virus BC-1 had inadequate coverage in the VP1 domain to get a good profile of SNVs across the domain, but some high-frequency mutations toward the 5′ end of the domain were observed. The VP1 domain of virus BC-2 had many SNVs distributed across the domain, at frequencies of 1 to 47%.

10.1128/mSphereDirect.00157-19.7 FIG S5 Environmental quasispecies of marine RNA viruses. The distribution of single-nucleotide variants (SNVs) along the helicase (A) and VP4 (B) domains of marine RNA viruses BC-1, BC-2, and BC-3, JP-A, JP-B, and Heterosigma akashiwo RNA virus (HaRNAV) is shown. Triangles (▲) indicate synonymous mutation variants, and circles (●) indicate nonsynonymous variants. The locations/samples where they were detected are illustrated in different colors. The SNV analysis was conducted using the quality-based variant detection tool of CLC Genomics Workbench. Download FIG S5, EPS file, 0.1 MB. Copyright © 2019 Vlok et al. 2019 Vlok et al. This content is distributed under the terms of the Creative Commons Attribution 4.0 International license.

### Detection of established viral variants.

**FIG 6:**
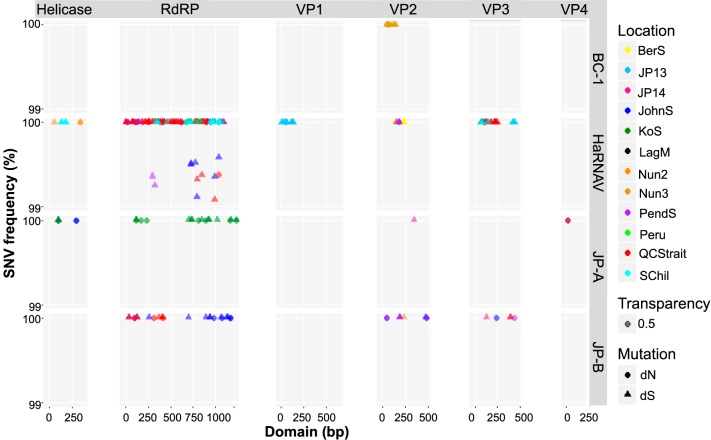
Established viral variants in different marine RNA virus communities. The distribution of high-frequency (>99%) single*-*nucleotide variants (SNVs) along the respective domains of the BC-1, HaRNAV, JP-A, and JP–B viruses is shown. Triangles (▲) indicate synonymous mutation variants, and circles (●) indicate nonsynonymous variants. The locations/samples where variants were detected are illustrated in different colors and include Bering Sea (BerS; yellow), Jericho Pier 13/14 (JP; blue/pink), Johnstone Strait (JohnS; royal blue), Kenton-on-Sea (KoS; green), Laguna Madre (LagM; black), Nunavut 2/3 (Nun; orange/brown), Pendrell Sound (PendS; purple), Peru (green), Queen Charlotte Strait (QCStrait; red), and South Chile (SChil; teal).

Single-nucleotide variants that were more frequent than 99% were abundant and detected at multiple locations (Fig. 6). These are variants from the original isolate/genome that have clearly become established in the current population and were most common in the RdRp domains. JP-B variants were detected in Queen Charlotte Strait (8 SNVs) and Johnstone Strait (15 SNVs), and variants of HaRNAV occurred at both of these sites (50 and 56 SNVs, respectively) and at Pendrell Sound (20 SNVs), South Chile (18 SNVs), Laguna Madre (28 SNVs), Bering Sea (10 SNVs), Jericho Pier 13 (10 SNVs), Nunavut 2/3 (33 and 43 SNVs, respectively), and Peru (7 SNVs). Note that the data for the latter four locations are partially obscured in Fig. 6. In contrast, high-frequency SNVs of JP-A were detected exclusively from Kenton-on-Sea (18 SNVs).

Unlike the RdRp domain, fewer high-frequency SNVs were associated with other domains, especially for the helicase, VP4, and VP1. SNVs that were concentrated in the same region of the VP3 domain of HaRNAV were identified in data from Bering Sea (3 SNVs), Jericho Pier 13 (2 SNVs), Johnstone Strait (6 SNVs), Laguna Madre (2 SNVs), Nunavut 2/3 (6 and 2 SNVs, respectively), Pendrell Sound (2 SNVs), Peru (3 SNVs), Queen Charlotte Strait (14 SNVs), and South Chile (4 SNVs).

Of the three new genomes, BC-1 was the only one in which high-frequency SNVs were identified; SNVs in the VP2 domain were detected from Nunavut 3 (11 SNVs). SNVs in the VP2 domain of HaRNAV were also detected at Nunavut 3 (4 SNVs), Bering Sea (9 SNVs), and Pendrell Sound (4 SNVs). Detection of SNVs in multiple domains of a single virus at specific locations supports the existence of biogeographical variations for the virus, with established genome variants existing in these locations.

## DISCUSSION

Metagenomic analysis uncovered sequences for three previously unknown marine picorna-like RNA viruses (BC-1, BC-2, BC-3). The distributions of these and three related viruses (JP-A, JP-B, HaRNAV) (23, 44) within global RNA virus metagenomic data sets revealed striking differences, even though the viruses originated from the same area. An analysis of sequence variation revealed that these viruses exist as quasispecies and that purifying selection is the predominant evolutionary process acting on their genomes. These observations and their implications are discussed in detail below.

### Discovery of three divergent marine picorna-like viruses.

Three nearly complete genomes (BC-1, -2, -3) were assembled from the Jericho Pier 14 sample (Fig. 1), adding to genomes for HaRNAV, JP-A, and JP-B that originated from water collected in the same area (23, 44). BC-2 and -3 are most genetically similar, and while BC-1 shares similarity with BC-2 and -3, it also has sequence similarity with JP-A. Among the six viruses, HaRNAV is the most distantly related to the others (Fig. 1; see Fig. S6 and S7 in the supplemental material), consistent with previous phylogenetic analyses of RdRp sequences that place HaRNAV as relatively divergent within marine picorna-like viruses (7). Most marine picorna-like virus isolates infect diatoms (*Bacillariophyta*), which are abundant, genetically diverse, and globally distributed. In contrast, the host of HaRNAV is Heterosigma akashiwo, a member of the *Raphidophyta*, a family comprised of relatively few and less diverse protists (46); HaRNAV is the only RNA virus isolated that infects a raphidophyte. Therefore, the greater genetic distance between HaRNAV and other marine picorna-like viruses probably reflects the lack of other viruses infecting raphidophytes.

10.1128/mSphereDirect.00157-19.8 FIG S6 Pairwise nucleotide sequence similarities and neighbor-joining trees of the conserved domains of six marine RNA viruses. The nucleotide sequences of the respective domains were aligned according to the corresponding amino acid sequence alignments and trimmed at the ends so that similar regions of all viruses were compared. The percent identities and Jukes-Cantor distances are displayed along with neighbor*-*joining trees for the trimmed helicase (A, B), RdRp (C, D), VP2 (E, F), VP4 (G, H), VP3 (I, J), and VP1 (K, L) domains. The percentage of trees in which the associated taxa clustered together is shown next to the branches (based on 1,000 bootstrap replicates). Darker shading indicates more closely related sequences. This indicates that the six viruses are all distantly related, with BC-2 and -3 comparatively more similar to one another and HaRNAV the most divergent of the six (Fig. 1). Of the six analyzed domains, the VP3 and VP1 structural domains were the least conserved among the genomes. Similar trends were observed in pairwise protein sequence similarity analyses (Fig. S7). Download FIG S6, EPS file, 0.3 MB. Copyright © 2019 Vlok et al. 2019 Vlok et al. This content is distributed under the terms of the Creative Commons Attribution 4.0 International license.

10.1128/mSphereDirect.00157-19.9 FIG S7 Pairwise amino acid sequence similarity and neighbor-joining trees of the conserved domains of the six analyzed marine RNA viruses. The respective amino acid sequences were aligned for each domain and trimmed at the edges so that the same regions of the domains were compared for all six viruses. The percent identities and Jukes-Cantor distances are displayed, along with neighbor-joining trees, for the trimmed helicase (A, B), RdRp (C, D), VP2 (E, F), VP4 (G, H), VP3 (I, J), and VP1 (K, L) domains of marine RNA viruses BC-1, BC-2, and BC-3, JP-A, JP-B, and Heterosigma akashiwo RNA virus (HaRNAV). Trees were obtained by applying neighbor-joining and BioNJ algorithms to a matrix of pairwise distances estimated using a JTT model. The percentages of trees in which the associated taxa clustered together are shown next to the branches (bootstrap, 1,000). Download FIG S7, EPS file, 0.3 MB. Copyright © 2019 Vlok et al. 2019 Vlok et al. This content is distributed under the terms of the Creative Commons Attribution 4.0 International license.

Sequence divergence is different among the viral sequence domains. In particular, the protease is most divergent, whereas VP1 is the most divergent among the conserved structural proteins (VP1, VP2, and VP3). This is congruent with read recruitment (Fig. 2), in which fewer reads were detected for the VP1 and protease domains. VP1 can be involved in host recognition (47), and the protease modifies the host proteome during infection (48, 49). Thus, both proteins are likely tightly coupled with the host and more sequence divergence in these domains would be expected among different viruses.

Although all metagenomic data sets were interrogated for complete genomes, the three new genomes originated from a single sample, Jericho Pier 14. This may be because Jericho Pier 14 was the newest sample and the RNA genomes may have been less degraded than in the older samples (Table S1), which were stored at 4°C as virus concentrates prior to processing.

10.1128/mSphereDirect.00157-19.1 TABLE S1 Environmental descriptions of stations from which viral metagenomes were generated. Download Table S1, DOCX file, 0.02 MB. Copyright © 2019 Vlok et al. 2019 Vlok et al. This content is distributed under the terms of the Creative Commons Attribution 4.0 International license.

### Marine RNA virus biogeography.

The six viruses were selected for this study because they were all discovered in waters adjacent to Vancouver. Despite the large number of reads from Jericho Pier 14 that recruited to the three new genomes (BC-1, -2, and -3), few reads recruited to these genomes from a sample (Jericho Pier 13) collected a year earlier from the same location (Fig. 2A). In contrast, many more reads recruited to HaRNAV from the Jericho Pier 13 sample than from the Jericho Pier 14 sample. This shows that the relative abundances of these viruses are dynamic at the Jericho Pier site and is consistent with differences observed in the relative abundances of specific RdRp sequences at this location and nearby (7, 50).

Reads mapping to the viral genomes were distributed across wide geographical and environmental distances. Large portions of the JP-A and JP-B genomes were covered by reads from Queen Charlotte Strait and Johnstone Strait (Fig. 2A), whereas the coverage was much less for the BC-2, BC-3, and HaRNAV genomes. The variability in relative coverage of the viral genomes among metagenomic data sets from coastal British Columbia emphasizes the dynamic nature of the RNA viral communities across space and time. It shows that despite the oceanographic connectivity among these sites and the high dispersal potential of marine viruses, environmental and host-driven factors are critical in dictating the composition of marine RNA virus assemblages. This is consistent with the influences of host traits and environmental conditions on virus distribution (16) and metacommunity analysis of microbial communities in the Arctic Ocean (51). For example, HaRNAV infects a widely distributed host (H. akashiwo) that is sporadic in occurrence (52–56), explaining the widespread distribution of sequences matching this virus. However, HaRNAV exhibits host strain specificity (4), which explains why high-identity sequences mapping to large portions of the genome were not found across all samples. In contrast, JP-A, BC-2, and BC-3 show high similarity to viruses infecting diatoms in the genus *Cheatoceros* (Fig. 1B), which are much more abundant worldwide, especially in temperate waters. Therefore, it is not surprising that the sequence coverage for these viruses is typically much greater than that for HaRNAV.

The widespread presence of low-identity reads in coastal waters across locations indicates that distant relatives of these six viruses are widely distributed (Fig. 2). The large number of low-identity sequences mapping to the highly conserved RdRp domains is also consistent with the large amount of genetic diversity observed in gene surveys of RdRp sequences (5–8, 57). However, our data for full genomes show that there is also widespread diversity associated with the helicase and capsid domains (Fig. S2). Although the RdRp domain is generally the most well-conserved domain, in many samples there was discordance among the domains with respect to the number of sequences detected. For example, for the JP-A virus, many sequences from the JP13 sample mapped to the helicase region (Fig. S3Q), whereas few were associated with the RdRp domain. This could reflect different evolutionary pressures acting on the two domains or that recombination has occurred, both of which have been observed in picornaviruses (58). Regardless of the mechanism of diversification, a high degree of sequence richness was observed in viruses related to the six investigated here that occurred across widely separated regions. In contrast to the other viruses, very few low-identity sequences mapped to HaRNAV, perhaps because this virus infects hosts that have relatively low species richness. Conversely, the high diversity of sequences related to those from viruses assembled from metagenomic data suggests that these sequences are from viruses that infect hosts with greater genetic diversity.

The freshwater metagenomes (Fig. 2B) provide context for interpreting the marine data sets. The same host species, and therefore the same viruses, are unlikely to occur in fresh and marine waters. For example, there are no known freshwater *Heterosigma* species, but there are freshwater raphidophytes. Therefore, the amino acid sequences with an inferred relatively low identity of <70% found in freshwater metagenomic data sets that match HaRNAV sequences likely represent distant relatives of this virus that infect other species of raphidophytes. Furthermore, read recruitment from freshwater streams (*n* = 14 months) emptying into the marine water body from which the BC viruses were assembled suggests that these viruses are not of freshwater origin, as very few, if any, reads could be recruited (unpublished data).

### Purifying selection acts on the marine RNA virus quasispecies.

RNA virus replication results in “clouds” of sequence variants referred to as quasispecies upon which selection operates. In turn, selection is often viewed through the lens of purifying or negative selection, or the extent to which deleterious alleles are selectively removed from the population. Elevated purifying selection is commonly inferred from lower ratios of nonsynonymous (*dN*) to synonymous changes (*dS*) of nucleotides in a genome. Our data imply that purifying selection acts on both structural and nonstructural domains of the virus genomes, with overall *dN*/*dS* values of <1 (Fig. 4), consistent with postulated purifying selection in the RdRp domain (40). Purifying selection constrains the possible amino acid substitutions, so that most nucleotide changes conserve the amino acid sequence, as frequently observed in RNA viruses (59–61). Purifying selection is evident in the domain alignments, in which many amino acids that are conserved among the six genomes are encoded by alternate codons (Fig. S8). The capsid VP1 domain is an exception (Table S2), suggesting that this domain may be important in host recognition, as discussed earlier, and would therefore differ among viral taxa based on their hosts’ receptors.

10.1128/mSphereDirect.00157-19.10 FIG S8 Nucleotide alignments of the RdRp, helicase, VP2, VP-4, VP-3, and VP-1 domains of marine RNA viruses BC-1, BC-2, and BC-3, JP-A, JP-B, and Heterosigma akashiwo RNA virus (HaRNAV). The nucleotide sequence alignments were constructed based on the corresponding amino acid sequence alignments, with the edges trimmed to remove regions not represented in all viruses. Download FIG S8, EPS file, 0.5 MB. Copyright © 2019 Vlok et al. 2019 Vlok et al. This content is distributed under the terms of the Creative Commons Attribution 4.0 International license.

10.1128/mSphereDirect.00157-19.2 TABLE S2 Stochastic dominance observed in the domain sample pairs, analyzed using the Conover-Iman test. Download Table S2, DOCX file, 0.01 MB. Copyright © 2019 Vlok et al. 2019 Vlok et al. This content is distributed under the terms of the Creative Commons Attribution 4.0 International license.

The existence of quasispecies was validated for the six viruses by examining single-nucleotide variants (SNVs) in the sequence data from different locations. Not surprisingly, because they were assembled from the Jericho Pier 14 samples, BC-1, -2, and -3 had the greatest genome coverage, yet BC-3 recruited about four times more data than BC-1 and -2 but fewer SNVs for the RdRp, helicase, VP2, and VP3 domains (Fig. 5A, B, and C; Fig. S5A).

If a population of viruses contains an abundance of the fittest genotype surrounded by rarer quasispecies variants, most genotypes should resemble the original virus that replicated. This suggests that BC-3 stemmed from a lytic event that occurred shortly before sample collection, resulting in higher abundance and lower diversity. Similarly, an analysis of quasispecies in an Antarctic lake revealed that an abundant RNA virus genotype, APLV1, had few SNVs, with most occurring in <10% of the sequences, despite the virus being ecologically successful (36).

The patterns in the SNVs in the environmental data that mapped to JP-A and HaRNAV indicated that quasispecies-like populations occurred at locations beyond the original site of discovery (Fig. 5A). For example, SNVs were detected across the RdRp domain for HaRNAV and JP-A at Queen Charlotte Strait and Johnstone Strait and for HaRNAV at Pendrell Sound. Very few reads from Nunavut, Laguna Madre, or Kenton-on-Sea mapped to these genomes with sufficient similarity to call SNVs across the RdRp domain, but the detected variants were all synonymous and exhibited different frequencies, with the frequency of the Kenton-on-Sea variants being much lower than that of the Nunavut and Laguna Madre variants. While it is tempting to hypothesize that quasispecies are broadly distributed, there were too few matching sequences from Nunavut, Laguna Madre, and Kenton-on-Sea to conclusively state that they are indicative of quasispecies. Realistically, it is more likely that these sequences correspond to similar viruses, some of which may not infect the same host.

For the BC-3 virus, only the VP1 domain had enough variation to suggest a possible quasispecies (Fig. 5D). This domain is probably important for binding to the host receptor (47, 62, 63); thus, virus-host coevolution would affect selection on this domain. For the VP1 from BC-3, three of the four SNVs detected in both Jericho Pier 14 libraries coded for amino acid changes. Similarly, more low-frequency nonsynonymous SNVs were found in the VP1 domains of BC-1 and BC-2 (Fig. 5D). Both functional and structural studies suggest that RNA viruses tolerate restricted numbers and types of mutations (31). For example, during large-population passages of foot-and-mouth disease virus (FMDV), numerous nonsynonymous mutations occurred in the capsid protein, with 96% being on the protein surface (64). Without structural homology models for the proteins, it is not known if the nonsynonymous mutations correspond to sites located on the outside of the virus particle. Overall, the observed differences, along with the higher *dN*/*dS* ratio previously discussed, suggest that VP1 domains experience different degrees of evolutionary pressure in comparison to the other capsid proteins, perhaps due to the role of VP1 in host recognition.

### Established viral variants are widely dispersed.

Given the coevolutionary arms race between viruses and their hosts, and the competitive advantage of existing as quasispecies, some low-frequency variants should be selected for and become established as the fittest genotype. This would result in turnovers in the dominant viral genotypes, as frequently occurs in viruses of multicellular organisms (32, 65, 66). To investigate how HaRNAV, JP-A, and JP-B have changed since they were first sequenced, the high-frequency SNVs (>99%) detected in the environmental data sets were analyzed. High-frequency SNVs in the RdRp of HaRNAV indicate that many of the established genetic variants occur at geographically distant locations, including Bering Sea, Johnstone Strait, Queen Charlotte Strait, Pendrell Sound, Laguna Madre, Peru, South Chile, and Nunavut 2/3 (Fig. 6). Similarly, high-frequency variants of JP-B were also detected at geographically distinct locations (Johnstone Strait and Queen Charlotte Strait), but unlike HaRNAV, where location-specific sequences were frequently found for more than one domain, the domains and locations were for the most part distinct for JP-B. A similar discord was observed for JP-A, where only the RdRp domain was covered (Fig. 6). Despite inconsistent coverage across genomes, geographically distinct established variants were detected for multiple genotypes, indicating that turnover in the dominant genotype occurs.

Most SNVs in the HaRNAV and JP-B genomes were synonymous, indicating pressure to conserve amino acid sequences. In contrast, the SNVs in the RdRp of JP-A were more equally distributed between synonymous and nonsynonymous. The JP-A RdRp sequences originated from samples collected at Kenton-on-Sea, on the southeast coast of South Africa, which is very distant from the coast of southwestern Canada, where the JP-A genome originated. The large proportion of nonsynonymous substitutions suggests that the Kenton-on-Sea variant is evolutionarily relatively distant from the JP-A genome, emphasizing that marine picorna-like viruses are diverse and widespread in the world’s oceans.

### Conclusions.

Assembly of marine RNA virus metagenomic data from a single metagenomic sample in British Columbia revealed three previously unknown viruses, BC-1, -2, and -3. Recruitment of sequences from 17 metagenomic data sets to these and other previously characterized marine RNA virus genomes uncovered biogeographic patterns and evidence for quasispecies and purifying selection in marine RNA viruses across distant locations.

The occurrence of genetically related viruses differed across oceans and hemispheres, implying that biotic factors such as virus life cycles and host traits, rather than solely abiotic factors, affected their distribution. In addition, differences in community composition across locations and years attest to the dynamic nature of marine RNA virus assemblages and are consistent with the idea that these dynamics probably result from episodic infections (7).

Numerous sequences with low amino acid identities to the six viruses were detected, revealing a great richness of globally distributed, distantly related viruses. Single-nucleotide variation was also observed in structural and nonstructural protein domains, consistent with purifying selection; thus, despite the error-prone RdRp and its lack of proofreading, these sequences are constrained to conserve their function. However, the VP1 domain had a statistically higher *dN*/*dS* ratio than the other domains, the highest distance scores in pairwise similarity analyses, and a unique “cloud” of high-amino-acid-identity sequences. These observations are congruent with the conjecture of VP1 being important in host receptor binding (47), leading to greater amino acid sequence variation in response to host evolution. Of the three new viruses, BC-3 had the greatest coverage and the fewest low-frequency SNVs in the metagenomic data, consistent with a recent lytic event that proliferated the dominant genotype. Detection of high-frequency SNVs for HaRNAV, JP-A, and JP-B indicates that these sequences are related to those from the originally identified viruses but that due to purifying selection, most of the mutations are synonymous. The RdRp sequences of the JP-A-like virus detected from Kenton-on-Sea exhibited many nonsynonymous mutations, indicating that distant relatives of this virus are widely distributed. Clearly, both distant and close relatives of the viruses presented here are distributed across oceans and hemispheres, implying a balance between dispersal and selection within the quasispecies cloud generated during RNA virus replication.

## MATERIALS AND METHODS

### Sample collection and preparation.

**FIG 7:**
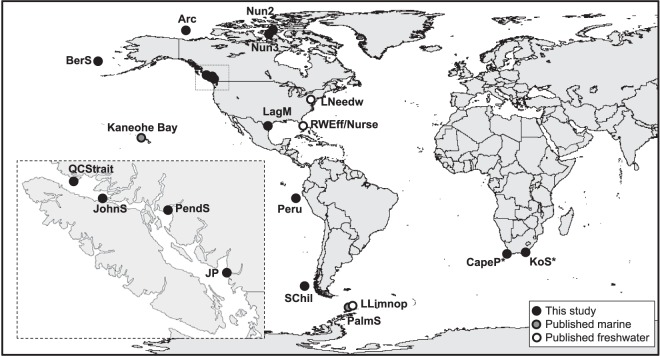
Sampling locations represented in this study. The samples represent multiple depths and time points from geographically distinct locations and ocean climate zones. Metagenomes from 14 locations were analyzed here (black) along with published marine data (gray) from Kaneohe Bay (KaneoB), Hawaii (57), and Palmer Station (PalmS), Antarctica (8), freshwater data sets (white) generated from samples from Lake Limnopolar (LLimnop), Antarctica (36), and Lake Needwood (LNeedw), USA (82), and reclaimed effluent and nursery water (RWEff and RWNurse) from Florida, USA (83). See the text for other location abbreviations.

Fourteen samples were processed from thirteen locations: four along the temperate southwest coast of Canada (Jericho Pier [JP], Johnstone Strait [JohnS], Pendrell Sound [PendS], and Queen Charlotte Strait [QCStrait]); one composite of 10 samples from the Canadian Arctic (Arc); two from the Nunavut coastal ocean (Nun2 and Nun3) representing the northern subpolar regions; one in the temperate/subpolar Bering Sea (BerS); one from Laguna Madre, representing the subtropical Gulf of Mexico (LagM); one from tropical coastal Peru (Peru); one from temperate southern Chile (SChil); and two from the coastal warm Indian Ocean Aghulas (Kenton-on-Sea [KoS]) and cold Southern Atlantic Benguela (Cape Town [CapeP]) currents of South Africa (Fig. 7). The samples were collected at various times (see Table S1 in the supplemental material), and JP was sampled in two consecutive years, 2013 (JP13) and 2014 (JP14). The JP14 sample was split in two and concentrated using different methods.

Samples were collected using either Niskin bottles mounted on a CTD rosette (∼150 to 200 liters) or by bucket from the surface (JP, KoS, CapeP) (∼2 to 20 liters). Particulate matter was removed by pressure filtering (<17 kPa) the water through glass fiber filters (MFS GC50; nominal pore size, 1.2 µm) and polyvinylidene difluoride filters (Millipore GVWP; pore size, 0.22 µm), after which viruses were concentrated using either ultrafiltration (67) through a 30-kDa-cutoff cartridge (Amicon S1Y30; Millipore) or chemical flocculation (68) with resuspension in ascorbate-EDTA buffer. Virus concentrates were stored at 4°C in the dark until processed for sequencing.

### cDNA synthesis and metagenomic library preparation.

Viruses (70 ml) were further concentrated by ultracentrifugation (120 000 × *g* for 5 h at 8°C), and each pellet was resuspended overnight at 4°C in 400 µl of supernatant liquid. Nucleic acids were extracted using the PureLink viral RNA/DNA mini kit (Thermo Fisher Scientific), and the DNA was removed using TURBO DNase (Thermo Fisher Scientific). Five replicates of cDNA were synthesized, with various concentrations of template RNA, by sequence-independent single-primer amplification (SISPA) using both random-hexamer (AD88, 5′-CCTGAATTCGGATCCTCCNNNNNN-3′) and nonamer (SMA-p1, 5′-GACATGTATCCGGATGTNNNNNNNNN-3′) primers (69, 70) and Superscript III reverse transcriptase (Thermo Fisher Scientific) per the manufacturer’s recommendations.

Amplification of cDNA was carried out in four replicates using primers AD89 (5′-CCTGAATTCGGATCCTCC-3′) and SMA-P2 (5′-GACATGTATCCGGATGT-3′) (69, 70) and Platinum *Taq* DNA polymerase (Thermo Fisher Scientific) per the manufacturer’s protocol, with the addition of 5% (vol/vol) dimethyl sulfoxide (DMSO) and various concentrations of MgCl_2_. All replicates were pooled, cleaned using the Genomic DNA Clean and Concentrator kit (Zymo), and sheared to approximately 300 bp using an S220 focused-ultrasonicator (Covaris).

Size selection (>200 bp) was done using Agencourt AMPure XP beads (Beckman Coulter), and libraries were prepared using NEXTFLEX-96 DNA barcodes (Bioo Scientific) and the NxSeq DNA sample prep kit (Lucigen) per the manufacturer’s protocol. Pooled libraries were sequenced on an Illumina HiSeq 2000 (2 × 100 bp) at the Biodiversity Research Centre, University of British Columbia, Vancouver, Canada.

### Assembly of metagenomic reads and genomic analysis.

Primer sequences were removed and quality trimmed (PHRED score of 30) using Trimmomatic (71). Paired reads were merged with PEAR (72) and combined with the reads that had no pairs, to produce the final data sets. Each metagenome was assembled separately using the default settings of the *de novo* assembly algorithm in the CLC Genomics Workbench v7.5 (CLCBio). The contigs and unassembled reads for each metagenome were combined and reassembled using the same parameters.

Open reading frames (ORFs) were identified using the CLC Genomics Workbench v7.5. ORFs smaller than 350 bp were not included in further analyses. The ORFs were compared to the Conserved Domain Database (CDD) (73) (October 2016) using BLASTp and the Pfam database (74) (December 2015) using HMMER v3.1b2 (75). Contig size, ORF architecture, and domain identity were used to identify nearly complete genomes. All predicted untranslated regions (UTRs) of assembled genomes were analyzed using IRESite (76) to identify potential internal ribosomal entry sites (IRES).

RdRp alignments were generated using MUSCLE v3.8.425 with the default parameters (77) and then manually refined with AliView version 1.17.1 (78). Amino acid model selection was conducted using the Smart Model Selection in PhyML (79). The maximum likelihood trees were constructed using the aLRT SH-like approach in PhyML 3.0 (80), the RtREV+G+I+F amino acid model with 100 bootstrap replicates. The tree was edited in iTOL v3 (81). Sequences used are deposited under accession numbers NP944776, BAE47143, YP001429583, AGZ83339, AFM44930, AHA44480, YP009047193, BAG30951, BAE79742, YP002647032, YP009230124, YP009230118, YP009230120, YP001429581, YP009111336, AYD68773, AYD68775, AYD68777, and BAK40203.

### Metagenomic read recruitment.

Reads were recruited from viral metagenomic data sets (Fig. 7; Table S1), either generated in this study or previously published (8, 36, 57, 82, 83), to the three newly assembled RNA genomes (MG584187, MG584188, and MG584189) and JP-A (NC_009757), JP-B (NC_009758), and HaRNAV (NC_005281) using tBLASTx (E value, 10^−10^). Conflicts were resolved based on the highest bit score and lowest E value. Translated reads were mapped onto the genomes based on percent amino acid identities and visualized with a 0.5 alpha setting to visualize overlapping reads using R 3.2.2 (84).

### Pairwise similarity and selection pressure analyses.

Predicted domain nucleotide and amino acid sequences were aligned using MUSCLE (77). Alignments were trimmed, by removing 5′ and 3′ ends that did not align, with AliView v1.17.1 (78). Pairwise sequence similarities were calculated and neighbor-joining trees were constructed using the CLC Genomics Workbench v7.5 and MEGA7 (85). The number of inferred synonymous (S) and nonsynonymous (N) substitutions for each codon were estimated using joint maximum likelihood reconstructions of ancestral states under a Muse-Gaut model of codon substitution (86) and the Felsenstein model of nucleotide substitutions (87), using Hyphy (88) and MEGA7. One-way analysis of variance (ANOVA), Kruskal-Wallis rank sum tests, and the Conover-Iman test, with Bonferroni corrections, were performed using base R 3.2.2 and the conover.test R package (89).

Metagenomic reads were mapped to the six genomes using the CLC Genomics Workbench v7.5 with a stringency of identity and read overlap of 90%. Single-nucleotide variation (SNV) analysis was conducted using the quality-based variant detection tool in CLC Genomics Workbench v7.5 (minimum read count of two).

### Accession number(s).

The newly assembled viral genomes are available from GenBank under the following accession numbers: MG584187, MG584188, and MG584189. Sequencing data were deposited in the BioSample database under the following numbers: SAMN08391118 to SAMN08391132.
