# Supplementary material for: Marine RNA Virus Quasispecies Are Distributed throughout the Oceans
Source: mSphere. 2019 Apr 3;4(2):e00157-19. doi: 10.1128/mSphereDirect.00157-19 (PMC6449609; doi:10.1128/mSphereDirect.00157-19)
Supplement: TABLE S1 [file mSphereDirect.00157-19-st001.docx]

| Code | Station | | TFF/ FeCl_2_ | Coordinates | | Salinity | Temp | Date Sampled | Sample age* |
| --- | --- | --- | --- | --- | --- | --- | --- | --- | --- |
|  |  |  |  | Latitude | Longtitude |  |  |  |  |
| E1 | Peru 97 | Peru | TFF | -8.7 | -85.2 | 32.3 | 26.8 | May-97 | 208 |
| E2 | Laguna Madre 94 | LagM | TFF | 27.5 | -97.3 | 36.1 | 30.0 | Jul-94 | 242 |
| E11 | Bering Sea a08 | BerS | TFF | 56.6 | -172.7 | 32.3 | 6.9 | Jul-08 | 74 |
| E34 | South Chile 97 | Schil | TFF | -50.9 | -80.8 | 33.8 | 7.8 | May-97 | 208 |
| E36 | Nunavut 2 | Nun2 | TFF | 70.7 | -98.6 | 31.9 | -1.0 | Jul-07 | 86 |
| E37 | Nunavut 3 | Nun3 | TFF | 71.9 | -94.3 | 30.4 | -0.8 | Jul-07 | 86 |
| MV1 | Arctic | Arc | TFF | 72.3 | -133.2 | 27.6 | 0.0 | Sep/Oct-02 | 144 |
| MV2 | Jericho Pier 14^+^ | JP14 | TFF | 49.3 | -123.2 | 23.3 | 10.8 | Apr-14 | 5 |
| MV3 | Jericho Pier 13 | JP13 | TFF | 49.3 | -123.2 | 27.0 | 9.4 | Apr-13 | 17 |
| MV4 | Johnstone Strait 11 | JohnS | TFF | 50.5 | -126.4 | 30.7 | 9.3 | Oct-11 | 35 |
| MV5 | Pendrell Sound 12 | PendS | TFF | 50.3 | -124.7 | 19.0 | 8.7 | Sep-12 | 24 |
| MV6 | Queen Charlotte Strait 11 | QCStrait | TFF | 50.8 | -127.1 | 31.0 | 8.9 | Oct-11 | 35 |
| MV7 | Jericho Pier 14^+^ | JP14 | FeCl_2_ | 49.3 | -123.2 | 23.3 | 10.8 | Apr-14 | 5 |
| MV8 | Kenton-on-Sea | K-o-S | FeCl_2_ | -33.7 | 26.7 | 35.0 | 20.5 | Dec-13 | 9 |
| MV9 | Cape Point | CapeP | FeCl_2_ | -33.9 | 18.4 | 30.0 | 15.9 | Dec-13 | 9 |

+ Virus concentrates were generated from the same sample which was split in two.

* Approximate number of months samples were stored at 4°C before RNA extraction.
