# Supplementary material for: Marine RNA Virus Quasispecies Are Distributed throughout the Oceans
Source: mSphere. 2019 Apr 3;4(2):e00157-19. doi: 10.1128/mSphereDirect.00157-19 (PMC6449609; doi:10.1128/mSphereDirect.00157-19)
Supplement: TABLE S2 [file mSphereDirect.00157-19-st002.docx]

| **Domain** | **Helicase** | **RdRp** | **VP1** | **VP2** | **VP3** |
| --- | --- | --- | --- | --- | --- |
| **RdRp** | 0.633432 |  |  |  |  |
|  | 1.0000^1^ |  |  |  |  |
| **VP1** | -1.624391 | -2.823822 |  |  |  |
|  | 0.7848 | 0.0364 |  |  |  |
| **VP2** | 0.890372 | 0.438220 | 2.760376 |  |  |
|  | 1.0000 | 1.0000 | 0.0442 |  |  |
| **VP3** | 0.369776 | -0.213719 | 2.125099 | -0.540363 |  |
|  | 1.0000 | 1.0000 | 0.2539 | 1.0000 |  |
| **VP4** | 1.294501 | 1.010319 | 2.575170 | 0.688016 | 1.052076 |
|  | 1.0000 | 1.0000 | 0.0763 | 1.0000 | 1.0000 |

^1^ Values obtained through comparison by groups were corrected by the Bonferroni method (shaded cells)
